# Integrated analysis of chronic lipotoxicity on muscle metabolism and stress and its reversal by antioxidants

**DOI:** 10.1186/2193-1801-3-251

**Published:** 2014-05-18

**Authors:** Mahesh Kumar Verma, Aggunda Nagaraju Yateesh, Rachapalli Smitha, Korrapati Neelima, Puttrevana M Pallavi, Madhusudhan Reddy, Jayaram Poornima, Anup M Oommen, Madanahalli R Jagannath, Baggavalli P Somesh

**Affiliations:** Connexios Life Sciences Private Ltd, No. 49, First Main road, 3rd phase, JP Nagar, Bangalore, 560 078 India

**Keywords:** Lipotoxicity, C2C12 myotubes, Glucose and fat metabolism, Oxidative capacity, Cellular stress, Antioxidant

## Abstract

Apart from elevated glucose, triglyceride and cholesterol, elevated levels of serum free-fatty acid (FFA) are observed in diabetic patients. Increased FFA load can cause multiple dysregulation which are collectively known as lipotoxicity. Impacts of FFA induced lipotoxicity were evaluated on various cellular responses of metabolism and stress in skeletal muscle myotubes. Under lipotoxicity, oxidative capacity of C2C12 myotubes was reduced and decreased levels ATP and NAD were observed. Lipotoxicity augmented non-oxidative disposal of metabolites in terms of lactate release, IMTG and ceramide synthesis. Concomitantly, insulin resistance was also observed. These impacts were in conjunction with increased cellular stress, inflammation, proteolysis and apoptosis. Quenching of lipotoxicity mediated oxidative stress by antioxidant reverted its deleterious impacts and restored insulin stimulated glucose uptake. In conclusion, the *in vitro* lipotoxicity makes a system which resembles *in vivo* pathology of muscle as seen in diabetic patients and represents an integrated perspective of lipotoxicity on various parameters of metabolism and stress.

## Background

Type-II diabetic patients are characterized by peripheral insulin resistance which hinders glucose uptake capacity of different organs and causes unregulated gluconeogenesis together resulting in hyperglycemia (DeFronzo et al. 1992). Unregulated rate of lipolysis is observed from insulin resistant adipose tissue (DeFronzo et al. 1992) which increases plasma free-fatty acid (FFA) levels. Elevated levels of plasma FFA are considered as a causal factor for insulin resistance (Itani et al. 2002) and reduction in serum FFA levels improves insulin sensitivity in humans (Qvigstad et al. 2003; Bajaj et al. 2004) as well as in animal models of disease (Ahrén 2001).

Skeletal muscle from T2DM patients shows reduced PI3K-Akt signaling and increased serine phosphorylation of IRS1 which inhibits insulin signaling (Kim et al. 1999; Krook et al. 2000; Bouzakri et al. 2003). Insulin induced GLUT4 translocation to plasma membrane followed by glucose uptake as well as glycogen storage capacity is reduced in skeletal muscle from diabetic patients (Kelley et al. 1996; Kim et al. 1999). Insulin resistance not only causes impaired glucose uptake and storage but also hinders overall oxidative capacity (Simoneau and Kelley 1997; Schrauwen and Hesselink 2004) as number and/or activity of mitochondria is reduced (Kelley et al. 2002; Barazzoni 2004; Asmann et al. 2006; Szendroedi et al. 2007). Moreover, it is evident that energy metabolism is altered and ATP production is reduced in diabetic muscles (Scheuermann-Freestone et al. 2003; Asmann et al. 2006; Szendroedi et al. 2007). Similarly, overall decreased or incomplete fat oxidation is observed in skeletal muscle from diabetic patients (Mootha et al. 2003; Patti et al. 2003; Mensink et al. 2007; Mogensen et al. 2007; Koves et al. 2008) despite having increased expression of genes involved in fat oxidation (Turner et al. 2007; Kulkarni et al. 2012). Besides defects in fat oxidation, increased fat uptake is observed in skeletal muscle which is another contributing factor for increased intramyocellular triglyceride (IMTG) storage (Bonen et al. 2004). In fact, IMTG levels can be directly correlated with insulin resistance in sedentary subjects (McGarry 2002). Interestingly, skeletal muscles from exercise trained subjects are insulin sensitive despite having high IMTG content (Goodpaster et al. 2001).

Apart from defects in metabolism, increased oxidative and endoplasmic reticulum (ER) stress, chronic low grade inflammation, increased proteolysis were observed in skeletal muscle which collectively attribute to insulin resistance (Wang et al. 2006; Abdul-Ghani et al. 2009; Hey-Mogensen et al. 2010). In fact, cellular stress such as oxidative stress and stress signaling play a central role for development of insulin resistance (Evans et al. 2002; Wei et al. 2008).

Skeletal muscle pathology under diabetes can be seen as a combination of multiple disease states such as insulin resistance, elevated oxidative stress, impaired glucose and fat metabolism. An *in vitro* system that systematically addresses all these major pathologies will be an elegant model to study molecular mechanism underpinning skeletal muscle pathophysiology. Here, we use chronic lipotoxic conditions comprising saturated free-fatty acid (palmitate) to study various parameters of metabolism and stress. The lipotoxic conditions resulted in increased fatty acid uptake and elevated IMTG levels as well as increased expression of genes associated with TG synthesis. We find that *in vitro* lipotoxic conditions resulted in impaired oxidative capacity, reduced mitochondrial number and cellular ATP levels. Non-oxidative disposal of glucose in the form of lactate was increased while glycogen storage was decreased. Increased insulin resistance, oxidative stress, ER stress, apoptosis, proteolysis and low grade inflammation were confirmed under chronic lipotoxic conditions. Thus, our data represent an integrated view of *in vitro* lipotoxic conditions mimicking muscle pathology as seen in T2DM patients. Furthermore, we exploited this system of chronic lipotoxicity to examine oxidative stress as a mechanism to impair muscle metabolism. We provide evidence that treatment with antioxidant, a known mechanism to counteract oxidative stress, led to reduction in ER stress, JNK phosphorylation, IMTG storage and enhanced oxidative capacity. Therefore, we conclude that oxidative stress is one of the key mechanisms for development of lipotoxicity and its neutralization could provide potential therapeutic advantage.

## Results

### Lipotoxic conditions lead to TG accumulation and increased fatty acid uptake

**Figure 1:**
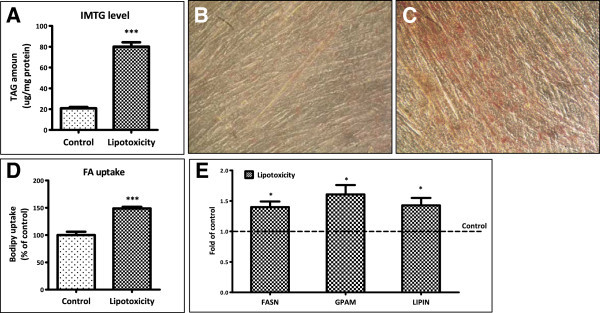
**Lipotoxic condition augments fat uptake and storage. (A)** C2C12 myotubes were treated with lipotoxic condition as described in “Methods” followed by quantitative estimation of triglycerides (*** P < 0.001). Oil-O-red staining showing TG levels in myotubes cultured under lipotoxic condition **(C)** or under control condition **(B)**. Fatty acid uptake capacity of myotubes **(D)** was measured by their ability to uptake a fluorescent fatty acid analog after chronic treatment (*** P < 0.001). After lipotoxic condition, RNA was isolated from myotubes and the mRNA levels of FASN, GPAM and LIPIN **(E)** (* P < 0.05) were measured as described in the Methods.

To explore the impact of lipotoxic conditions on muscle metabolism, we treated differentiated C2C12 myotubes with 750 μM of palmitate (Watt et al. 2012) for 16 h as described in Methods. Under this lipotoxic condition, we did not observe any appreciable cytotoxic impact (data not shown). To check whether this condition mimicked diabetic muscle pathology, we measured intramyocellular triglyceride (IMTG) levels. IMTG was found to be increased in myotubes cultured under lipotoxic conditions (~4 fold) as compared to those cultured under control condition (Figure 1A). Increased IMTG levels under lipotoxicity were also reflected in oil-o-red staining (Figure 1B, C). This increase in IMTG was accompanied by increased fatty acid uptake which was 49% higher under lipotoxic conditions (Figure 1D). To test whether lipotoxic conditions increased TG synthesis capacity of myotubes, we measured expression of genes involved in TG synthesis. We observed a significant increase in expression of genes required for fatty acid synthesis (FASN, 1.4 fold) and TG synthesis from fatty acids (GPAM, 1.6 fold and LIPIN, 1.4 fold) under lipotoxic conditions (Figure 1E). Hence, fatty acid uptake, TG synthesis and IMTG storage were increased in skeletal muscle myotubes under lipotoxic conditions.

### Attenuated fatty acid oxidation and mitochondrial biogenesis under lipotoxic conditions

**Figure 2:**
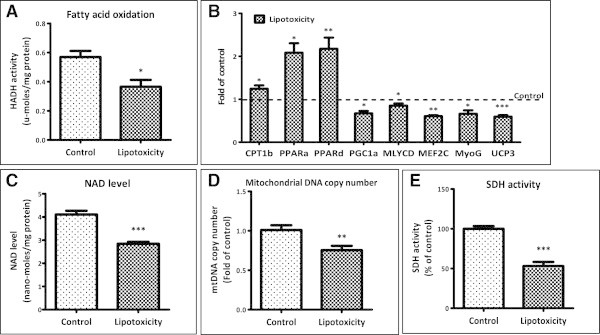
**Lipotoxicity impairs oxidative capacity of myotubes. (A)** Fatty acid oxidation ability was measured by HADH activity in lysates of myotubes cultured under control or lipotoxic condition (* P < 0.05). **(B)** Post lipotoxic condition, mRNA levels of mentioned genes were measured as described in the Methods (* P < 0.05, ** P < 0.01, *** P < 0.001). **(C)** Cellular NAD levels were quantified after lipotoxic treatment (*** P < 0.001). **(D)** Total DNA was isolated from myotubes cultured under control or lipotoxic condition and mitochondrial DNA copy number **(D)** was estimated by assessing mtND1 (mitochondrial gene) copy number normalized to HPRT (nuclear gene) (** P < 0.01). **(E)** SDH activity in myotubes cultured under control or lipotoxic condition (*** P < 0.001).

Next we studied the impact of lipotoxicity on fatty acid oxidation by estimating the activity of 3-hydroxyacyl-CoA dehydrogenase (HADH). Lipotoxic conditions reduced fatty acid oxidation as activity of HADH was decreased by 35% (Figure 2A). We also measured the expression of genes implicated for fat oxidation and energy metabolism. A decrease in expression of PGC1α (33%), MLYCD (15%) and UCP3 (40%) was detected in myotubes under lipotoxic condition compared to control condition (Figure 2B). Similarly expression of muscle enriched genes like MEF2C and MyoG were reduced by 39% and 34% respectively under lipotoxicity (Figure 2B). Interestingly, we observed an increase in expression of CPT1β (24%), PPARα (109%) and PPARδ (118%) under lipotoxic condition (Figure 2B). PGC1α is required for fat oxidation and uncoupling genes expression and is activated by deacetylation. Since, deacetylation process requires NAD, we next measure NAD levels in myotubes under lipotoxicity. Approximately 30% reduction in NAD level was observed under lipotocxicity (Figure 2C). In parallel with decrease in PGC1α and NAD levels, we observed a 24% decrease in mitochondrial DNA copy numbers in skeletal muscle myotubes under chronic lipotoxic condition (Figure 2D). In consistent with these findings, enzymatic activity of SDH (succinate dehydrogenase) was found to be reduced (53% of control, Figure 2E). Taken together, lipotoxicity severely impacts mitochondrial number and activity, muscle fat oxidation and redox balance.

### Lipotoxicity negatively impact muscle glucose metabolism

**Figure 3:**
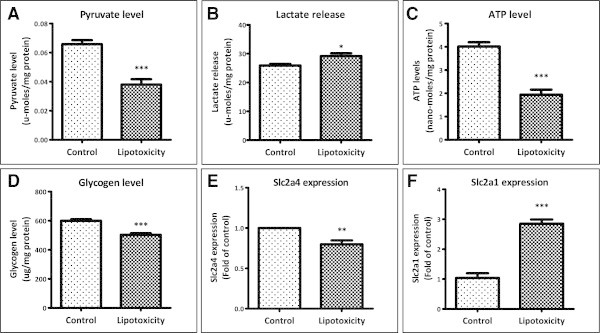
**Impact of lipotoxic condition on glucose metabolism. (A)** After lipotoxic treatment, myotubes were lysed and cellular pyruvate levels were measured (*** P < 0.001). **(B)** Lactate release from myotubes was measured in culture medium collected after lipotoxic treatment (* P < 0.05). **(C)** Cellular ATP levels (*** P < 0.001) and **(D)** glycogen storage (*** P < 0.001) were measured after lipotoxic treatment as described in Methods. After lipotoxic treatment, RNA was isolated and mRNA levels of glucose transporter Glut4/Slc2a4 (** P < 0.01; **E**) and Glut1/Slc2a1 (*** P < 0.001; **F**) were quantified using qPCR.

We investigated impact of lipotoxicity on glucose metabolism and found that pyruvate levels were decreased by 42% (Figure 3A) indicating glycolysis was reduced. Moreover, a significant increase (12%) in lactate release (29.1 μmoles/mg under lipotoxicity versus 25.9 μmoles/mg under control) in culture medium was observed (Figure 3B) which denotes increased non oxidative disposal of glucose. In conjunction with reduced oxidation and increased non-oxidative disposal, reduced levels of ATP were detected (1.9 nano-moles/mg under lipotoxicity versus 4 nano-moles/mg under control; Figure 3C) indicating overall attenuation in energy metabolism. Interestingly, non-oxidative disposal of glucose in form of glycogen storage was decreased (~16%, 503 μg/mg versus 600 μg/mg under control; Figure 3D). Expression of glucose transporter Glut4 (Slc2a4) was reduced around 20% (Figure 3E) whereas expression of basal glucose transporter Glut1/Slc2a1 was found to be up-regulated by 2.8 fold under lipotoxicity (Figure 3F). Thus, lipotoxicity significantly impaired muscle glucose metabolism.

### Lipotoxicity causes insulin resistance in myotubes

**Figure 4:**
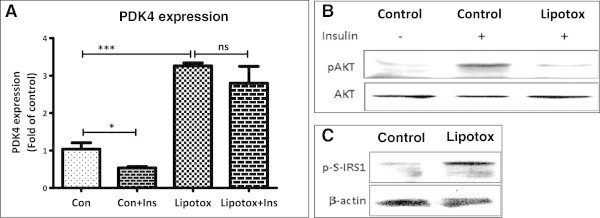
**Increased insulin resistance under lipotoxic condition. (A)** Post chronic lipotoxic treatment, myotubes were treated for 2 h with or without insulin and gene expression of PDK4 was analyzed (* P < 0.05, *** P < 0.001, ns: not significant). (Con: Control; Lipotox: Lipotoxicity; Ins: Insulin) **(B)** After chronic lipotoxic treatment, myotubes were serum starved and then treated with or without insulin for 10 min followed by analysis of pAKT and AKT levels by Western blotting. **(C)** After chronic lipotoxic conditions, myotubes were harvested for p-serine-IRS1 level measurement by Western blotting. Beta actin was used as a loading control.

We next studied the impact of lipotoxicity on muscle insulin resistance. Lipotoxic conditions caused an up-regulation of PDK4 expression (3.3 fold; Figure 4A), a gene inversely regulates glucose oxidation. In control myotubes, insulin treatment significantly reduced PDK4 expression (0.54 fold) implying insulin sensitivity and insulin mediated augmentation in glucose metabolism. However, insulin treatment failed to reduce PDK4 expression in myotubes cultured under lipotoxic condition confirming an insulin resistant state (Figure 4A). Acute treatment of insulin was able to increase Akt phosphorylation in control myotubes but not in those cultured under chronic lipotoxic conditions (Figure 4B). Furthermore, serine phosphorylation of IRS1, a marker of insulin resistance, was increased under lipotoxicity (Figure 4C). Overall, we conclude that lipotoxicity causes insulin resistance and hence decreases muscle glucose utilization.

### Chronic lipotoxic conditions elevate oxidative stress and ER stress

**Figure 5:**
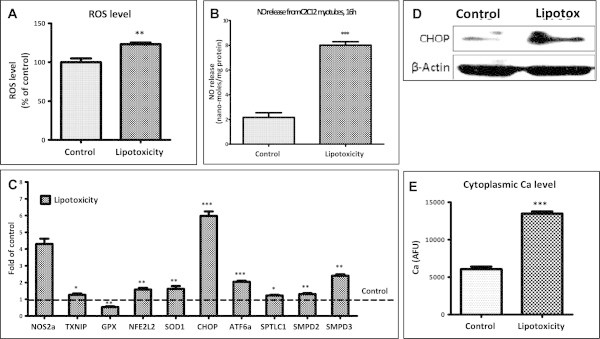
**Lipotoxicity causes oxidative stress, endoplasmic reticulum stress and ceramide synthesis in myotubes. (A)** Post lipotoxicity, myotubes were loaded with DCFH-DA ROS indicator probe and amount of ROS was quantified (** P < 0.01). **(B)** Nitric oxide levels in culture medium was quantified using Griess reagent (*** P < 0.001). **(C)** Expression of genes involved in oxidative stress, ER stress and ceramide synthesis in myotubes cultured under lipotoxic condition were quantified by real time RT-PCR and expressed as fold levels of control (Broken line) (* P < 0.05, ** P < 0.01, *** P < 0.001). **(D)** After chronic lipotoxic conditions, myotubes were harvested for CHOP level measurement by Western blotting. Beta actin was used as a loading control. **(E)** For estimation of cytoplasmic calcium levels, myotubes cultured under control or lipotoxic condition were loaded with Fluo-4 AM calcium indicator dye and the change in fluorescence was estimated as described in the Methods.

Chronic lipotoxic conditions increased oxidative stress in myotubes as ROS levels were increased by 20% (Figure 5A). Increase in ROS under lipotoxic conditions was accompanied with increase in nitric oxide levels (Figure 5B) which were enhanced by approximately 4 fold. We found that expression of GPX (glutathione peroxidase), an enzyme required to counteract ROS, was down-regulated under lipotoxicity (0.53 fold of control; Figure 5C). Expression of SOD1, NFE2L2/NRF2 and TXNIP – proteins which sense increased ROS levels, were up-regulated by 1.6, 1.6 and 1.3 fold respectively (Figure 5C). Similarly, expression of NOS2a/iNOS, an enzyme required for nitric oxide synthesis, was also up-regulated under lipotoxicity (4.3 fold of control; Figure 5C). Hence, oxidative stress is significantly augmented under lipotoxic conditions.

Next, we examined impact of lipotoxicity on ER stress. Both expression (Figure 5C) and protein levels (Figure 5D) of CHOP/DDIT3 (a marker of ER stress) were significantly increased under lipotoxic conditions. Similarly, ATF6α (another marker of ER stress) was also found to be up-regulated under lipotoxic conditions (2 fold of control; Figure 5C). In parallel to CHOP and ATF6α levels, cytoplasmic calcium levels were almost two times higher in myotubes cultured under chronic lipotoxic conditions compared to control myotubes (Figure 5E). Since oxidative stress and ER stress in muscle are linked to increased ceramide level, and expression of SPTLC1 (rate limiting enzyme for *de novo* ceramide biosynthesis), SMPD2 and SMPD3 (enzymes required for ceramide synthesis from sphingomyelin) were up-regulated under lipotoxicity (1.2, 1.3 and 2.4 fold respectively; Figure 5C). Taken together, we conclude that lipotoxic conditions increase muscle oxidative and ER stress with potential to enhance ceramide biosynthesis pathways.

### Augmentation of inflammation and inflammatory signaling under lipotoxic conditions

**Figure 6:**
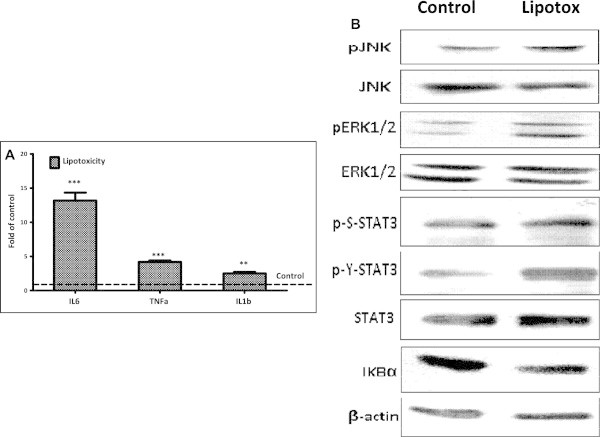
**Increased expression of pro-inflammatory cytokines genes and elevated stress signaling under lipotoxicity. (A)** Analysis of inflammatory cytokines mRNA levels in myotubes cultured under lipotoxic condition were quantified by real time RT-PCR and expressed as fold levels of control (Broken line) (** P < 0.01, *** P < 0.001). **(B)** Total cellular lysate from myotubes cultured under control or lipotoxic conditions was used for Western blotting of proteins as indicated.

We next ascertained whether chronic lipotoxicity can cause chronic inflammation in skeletal muscle. Skeletal muscle myotubes expressed significantly higher levels of pro-inflammatory myokines or cytokines such as IL6, TNFα and IL1β (Figure 6A) when cultured under lipotoxicity. This increase in inflammation in system led to an increase in NFκB signaling as IκB levels were found to be decreased (Figure 6B). Similarly JNK, ERK and STAT3 signaling were also found to be increased under lipotoxicity as we observed increased phosphorylated levels of these proteins (Figure 6B).

### Chronic lipotoxic conditions increases apoptosis and proteolysis

**Figure 7:**
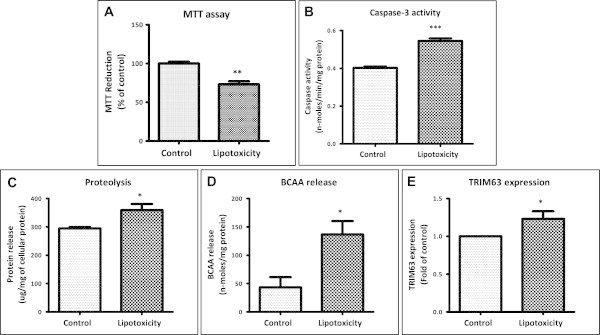
**Lipotoxic condition causes increased apoptosis and proteolysis in myotubes**. After chronic lipotoxicity, MTT assay (**A**, ** P < 0.01) and caspase-3 activity assay (**B**, *** P < 0.001) were performed as described under Methods. Proteolysis was estimated by elevated release of proteins (**C**, * P < 0.05) and branched chain amino acids (BCAA) (**D**, * P < 0.05) form myotubes into culture medium and up-regulated expression of proteolysis marker Trim63 (**E**, *P < 0.05).

We observed that cell survival is decreased under lipotoxic conditions (73% of control, Figure 7A) with a concomitant increase in Caspase-3 activity (0.55 nano-moles/min/mg versus 0.40 nano-moles/min/mg under control, Figure 7B), an indicator of apoptosis. Releases of proteins in culture medium (Figure 7C) as well as release of branched chain amino acids (3.2 fold, 137 nano-moles/mg versus 43 nano-moles/mg, Figure 7D) were increased from myotubes cultured under chronic lipotoxic conditions. Similarly, expression of TRIM63/MURF, a marker of proteolysis, was up-regulated under lipotoxicity (1.2 fold of control; Figure 7E). Hence, our data confirm that muscle apoptosis and proteolysis are increased by lipotoxic conditions.

### Antioxidant mediated reversal of lipotoxic impact in skeletal muscle myotubes

**Figure 8:**
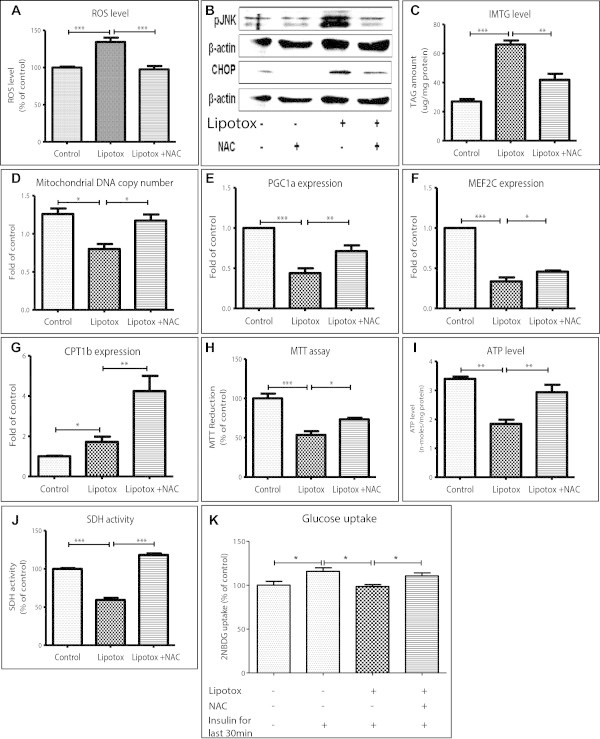
**Scavenging oxidative stress protects myotubes from lipotoxicity**. Myotubes were cultured under control or lipotoxic conditions or lipotoxic conditions containing N-acetyl cysteine (NAC). NAC, a ROS scavenger, reduced lipotoxicity induced ROS levels in myotubes (**A**, *** P < 0.001). **(B)** Stress signaling as measured by JNK phosphorylation and ER stress as measured by CHOP levels were reduced by NAC. **(C)** This antioxidant also reduced IMTG levels (*** P < 0.001; ** P < 0.01) and increased mitochondrial DNA copy number (**D**, * P < 0.05). Expression of PGC1α **(E)**, MEF2C **(F)** and CPT1β **(G)** genes was found to be increased by NAC (* P < 0.05, ** P < 0.01, *** P < 0.001). NAC treatment also increased cellular viability (**H**, *** P < 0.001; * P < 0.05) and ATP levels (**I**, ** P < 0.01). **(J)** Lipotoxic conditions decreased and NAC treatment restored SDH activity (*** P < 0.001). **(K)** Insulin stimulated glucose uptake was decreased under lipotoxic conditions and was restored by NAC treatment (* P < 0.05). ANOVA with Newman-Keuls post test was performed for statistical analyses.

We next studied the possibility of using chronic lipotoxicity system to validate potential molecular mechanism for their impact on diabetic muscle. We targeted oxidative stress using an antioxidant NAC (N-acetyl-L-cysteine) under chronic lipotoxic conditions and studied its impact on muscle stress and metabolism. As expected, being an antioxidant, NAC reduced lipotoxicity induced oxidative stress in muscle as ROS levels were decreased (Figure 8A). Reduction in oxidative stress was attendant with reduced inflammatory stress signaling and ER stress as JNK phosphorylation and CHOP levels were reduced (Figure 8B). Reduction in cellular stress by NAC resulted in improved metabolism which was observed with decreased IMTG storage (Figure 8C), increased mitochondrial biogenesis (Figure 8D) along with up-regulation of PGC1α, CPT1β and MEF2C expression (Figure 8E-G) under lipotoxic conditions. This improvement in metabolism also led to increased cell survival (Figure 8H) and also increased ATP levels (Figure 8I) indicating improvement in energy metabolism. Moreover, oxidative capacity, as measured by SDH activity, was reduced under lipotoxic conditions (59% of control) and was restored by antioxidant treatment (Figure 8J). Insulin stimulated glucose uptake was reduced under lipotoxic conditions indicating development of insulin resistance in myotubes which was restored by NAC treatment indicating reduction in oxidative stress can improve insulin sensitivity (Figure 8K).

In conclusion, our data provide first integrated analysis of dysregulation of various cellular responses under chronic lipotoxic conditions. This system mimics cellular pathology at various cellular responses as seen in skeletal muscle of T2DM patients and hence provides a useful tool for studying molecular mechanism or novel drug screening. Furthermore, we validated this system for its efficiency by targeting a known molecular mechanism to overcome lipotoxic effects on stress and metabolism.

## Discussion

This study presents integrated view of lipotoxicity induced gross impairment in various cellular responses of skeletal muscle. We used fatty acid overload as condition for inducing lipotoxicity as it is unambiguously a condition which is present in insulin resistant and T2DM patients (Fraze et al. 1985; Chen et al. 1987; Golay et al. 1987; Swislocki et al. 1987; Reaven et al. 1998), and found that all cellular and molecular parameters studied in this paper are impaired in accordance with that seen *in vivo* conditions. No appreciable cytotoxicity was observed under the lipotoxic condition which is consistent with the earlier reports (Sawada et al. 2012; Feng et al. 2012). Increased fatty acid levels exert toxic impacts, generally known as lipotoxic impacts, on a variety of cell types. We have earlier shown that saturated fatty acid, palmitate, is an important factor in deteriorating pancreatic beta cells’ health and function (Somesh et al. 2013). In skeletal muscle, it has been shown that palmitate treatment can cause inhibition of insulin signaling and glucose uptake as well as damage to mitochondria (Jové et al. 2006; Rachek et al. 2007; Yuzefovych et al. 2010). Palmitate treatment can also increase inflammatory cytokines gene expression and downstream signaling, oxidative stress and ceramide synthesis (Schmitz-Peiffer et al. 1999; Chavez et al. 2003; Jové et al. 2006; Rachek et al. 2007; Yuzefovych et al. 2010).

Lipotoxicity of skeletal muscle is defined as fatty acid overload mediated impairment in metabolism and increased stress. Reduced oxidative capacity, metabolic inflexibility and non-oxidative disposal of metabolites are characteristics of muscle of T2DM (Simoneau and Kelley 1997; He et al. 2001; Schrauwen and Hesselink 2004; Kim et al. 2006; Tsintzas et al. 2007). In this study, we are able to capture these deleterious impacts on metabolism like metabolic inflexibility or reduced ability of muscle to switch fuel from fat to glucose as seen in insulin resistant patients (Mensink et al. 2007). During insulin resistance, insulin mediated glucose oxidation comes down (Mensink et al. 2007) and hence fat becomes primary fuel source albeit overall oxidation is reduced. Our *in vitro* lipotoxic system is in agreement with these findings that fat oxidation is predominant form of oxidation under lipotoxic condition whereas glucose oxidation is concomitantly reduced. We provide evidence that under lipotoxic conditions expression of key genes regulating fat oxidation such as CPT1β, PPARα and PPARδ was increased while an increase in the expression of PDK4, a negative regulator of glucose oxidation, was observed. Our study also provide evidence that lipotoxic muscle are in a state of metabolic inflexibility and link this state to insulin resistance given that insulin treatment failed to reduce PDK4 expression in myotubes cultured under lipotoxic condition. This inability to switch fuel utilization from fat to glucose is seen in insulin resistance muscle as PDK4 expression remain elevated in response to insulin but is reduced in insulin sensitive muscles (Kim et al. 2006; Tsintzas et al. 2007; Kulkarni et al. 2012).

Though fat oxidation is predominant form of oxidation in insulin resistant muscle but overall oxidation is decreased or incomplete oxidation takes place (Koves et al. 2008). Fat oxidation and oxidative capacity are reduced in human patients (Kim et al. 2000; He et al. 2001; Oberbach et al. 2006) with a concomitant decrease in PGC1α expression and its downstream targets (Mootha et al. 2003; Patti et al. 2003; Debard et al. 2004). Our *in vitro* data are in agreement with these clinical findings as we report reduced fat oxidation under lipotoxic conditions along with reduced PGC1α level with concomitant decrease in mitochondria number and activity. Moreover, we show that ATP levels are also decreased indicating reduced overall oxidation. In parallel, we also provide evidence that NAD levels are decreased under lipotoxic conditions and this reducing agent is required for fuel oxidation hence further validating severely compromised oxidative capacity. Moreover, NAD is also required for SIRT1 protein mediated deacetylation and activation of PGC1α. Hence, it can be speculated that FFA overload to an extent higher than that can be handled by muscle causes lipotoxic impacts.

Packing of FFA into neutral lipid provides a possible mechanism to refrain from its toxic impacts (Listenberger et al. 2003). Consistent with this, we found increased IMTG levels and up-regulation of enzyme involved in TG packing under *in vitro* lipotoxicity. However, our data suggest that this protective mechanism was clearly not enough to completely protect myotubes from lipotoxic impacts. In this regards, activation of any other mechanism like increasing desaturation of fatty acid which aid TG packing might help better protection against lipotoxicity. This might be one of a reason that unsaturated fatty acid, oleate, does not cause lipotoxicity (Listenberger et al. 2003; Henique et al. 2010; Yuzefovych et al. 2010) as unsaturated fatty acids are preferred substrate for TG formation and incubation of myotubes with oleate leads to higher IMTG levels than those achieved with saturated fatty acid at same concentration (data not shown).

Increased fatty acid overload, uncontrolled oxidation and synthesis of complex lipids such as ceramide lead to increased cellular stress (Rachek et al. 2007; Yuzefovych et al. 2010). Saturated fatty acid can also activate NFkB signaling and produce pro-inflammatory cytokines (Jové et al. 2006). Increase in cellular stress and inflammation lead to mitochondrial dysfunction (Bonnard et al. 2008) thereby further aggravating lipotoxicity. Thus, cellular stress namely oxidative and ER stress, chronic low grade inflammation and downstream stress signaling become driver for the pathology of T2DM (Evans et al. 2002). Our *in vitro* system of lipotoxicity also supports these findings as we observe inflammation, oxidative and ER stress and stress signaling.

Since previous findings showed role of cellular ROS in inducing dysfunction of muscle and other cell types (Oprescu et al. 2007; Nakamura et al. 2009; Yuzefovych et al. 2010), we studied whether targeting oxidative stress can provide protection against lipotoxicity mediated impairments. Antioxidant mediated reduction in cellular oxidative stress led to a reduction in stress signaling and improved metabolic function of muscle cells under lipotoxic conditions. Antioxidant treatment improved oxidative capacity and mitochondrial functions of myotubes which led to efficient fat oxidation (data not shown) and reduced IMTG storage. These findings are in consistent with earlier reports showing that increasing muscle fatty acid oxidation capacity can protect from insulin resistance (Perdomo et al. 2004; Sebastián et al. 2007; Henique et al. 2010). Hence, any intervention capable of augmenting oxidative capacity and reducing oxidative stress could be of therapeutic importance.

## Conclusions

In this study, we provide an integrated perspective of lipotoxicity on skeletal muscle glucose and fat metabolism along with stress, inflammation and stress signaling. This integrated study can help to determine interrelation of different cellular machinery. Moreover, this *in vitro* system mimics muscle pathology as seen in T2DM patients and hence provides a reliable system to study disease mechanism or therapeutic interventions. Lastly, we target one cellular response of oxidative stress and show that this could be a potential mechanism to alleviate cellular stress and improve metabolic function of muscle.

## Methods

### Cell culture and induction of lipotoxicity

C2C12 myoblast cell line was procured from ATCC. The cells were maintained in undifferentiated state at low confluency in DMEM (Sigma) containing 25 mM glucose, 10% fetal bovine serum (Invitrogen), penicillin and streptomycin. For differentiation into myotubes, cells were seeded in multi-well plates and grown till complete confluency in growth medium and then cultured in reduced serum (2%) medium for next four days with medium renewal at every alternate day. After four days of differentiation, long multi-nucleated myotubes were seen and these differentiated cells were used for all experimentation.

Palmitate (Sigma) was dissolved in 50% ethanol and then conjugated in 10% BSA to get final concentration of 5 mM. For induction of lipotoxicity, differentiated myotubes were cultured with BSA conjugated palmitate (750 μM) for 16 h (Watt et al. 2012). Control myotubes were cultured with DMEM containing equal amount of BSA and ethanol.

For some experiments as indicated, myotubes were cultured under lipotoxicity in presence or absence of 5 mM N-acetyl-L-cysteine (NAC) (Sigma) for 16 h.

### Estimation of IMTG and oil-O-red staining

After inducing lipotoxicity for 16 h, myotubes were lysed and clear lysate was used for TG estimation using a commercially available kit (DiaSys). IMTG levels were normalized to cellular proteins measured using Bradford reagent (Bio-Rad). For oil-o-red staining, myotubes were fixed with 4% formaldehyde and then incubated for 15 min with 0.7% oil-o-red in 60% isopropanol. Cells were washed thrice and images were captured at 10× magnification.

### Measurement of fatty-acid uptake

Myotubes were washed with PBS and then incubated for 10 min with 1 μM of BODIPY dye (Molecular Probes), a fluorescent analog of fatty acid. Cells were washed to remove unbound dye and then excess dye was quenched with 0.4% trypan blue for 5 min. Myotubes were lysed and BODIPY fluorescent was estimated at 485 nm excitation/528 nm emission. Bodipy fluorescent was normalized to total cellular proteins and represented as % of vehicle control.

### Analysis of gene expression

Differentiated myotubes were cultured under control or lipotoxic conditions for 16 h. Post 16 h, total RNA was extracted using Tri reagent (Sigma) and converted into cDNA with reverse transcriptase and random hexamer primers (Applied Biosystems) using manufacturer’s protocol. This cDNA (5 ng per reaction) was used for quantification of gene expression using SYBR Green PCR Master Mix (Eurogenetic, Belgium). Genes analyzed in this study were FASN, GPAM, LIPIN, CPT1β, UCP3, MLYCD, MyoG, MEF2C, PPARα, PPARδ, PGC1α, Slc2a4, Slc2a1, PDK4, NOS2a, SOD1, GPX, NFE2L2, TXNIP, CHOP, SPTLC1, SMPD2, SMPD3, ATF6α, Trim63, TNFα, IL1β and IL6. β-actin or 18S rRNA was used as a housekeeping gene control.

For PDK4 (pyruvate dehydrogenase kinase 4) expression, myotubes were washed after chronic lipotoxic treatment and then incubated in serum and glucose free medium for 1 h. Subsequently, myotubes were treated with insulin (100 nM) for 2 h and then harvested for gene expression analysis.

### Measurement of 3-hydroxyacyl-CoA dehydrogenase (HADH) activity

To measure beta-oxidation of fatty acids, HADH enzyme activity was measured. Cell lysates were incubated with acetoacetyl-CoA and NADH for 1 h. Reduction in NADH level was measured at 340 nm. HADH activity was normalized to cellular protein.

### Estimation of cellular NAD, Pyruvate and ATP levels

NAD, Pyruvate and ATP levels were estimated in cell lysate using commercially available kits (EnzyChrom NAD+/NADH Assay Kit, BioAssay Systems; EnzyChrom Pyruvate Assay Kit, BioAssay Systems; ATP determination kit, Invitrogen) as per manufacturer’s procotol and were normalized to cellular protein.

### Mitochondrial DNA copy number

After chronic lipotoxic treatment, total DNA (5 ng) was used for quantitative real time PCR using a primer pair specific to mitochondrial DNA (ND1). Mitochondrial DNA copy number was normalized to nuclear DNA which was amplified using a primer pair specific to genomic DNA (HPRT).

### Estimation of succinate dehydrogenase (SDH) activity

After chronic lipotoxic treatment, myotubes were incubated with KRBH containing 50 mM sodium succinate, 50 mM sucrose, 1 mM sodium azide and 0.8 mM INT dye (p-Iodonitrotetrazolium Violet) for 30 min. For each treatment a separate set was also kept as blank which did not include sodium succinate. After 30 min, INT-Formazan formed due to SDH activity was dissolved in DMSO and measured at OD640. SDH activity was represented as % of vehicle control.

### Estimation of lactate release

Lactate release was measured in cell culture supernatant. After chronic lipotoxic treatment, cell culture medium was collected and centrifuged to remove debris. Lactate levels were estimated using EnzyChrom Lactate Assay Kit (BioAssay Systems) and normalized to total cellular protein.

### Measurement of glycogen levels

After chronic lipotoxic treatment, myotubes were lysed and amount of protein was measured. Lysates were treated with KOH solution (30%) at 95°C for 10 min followed by precipitation of glycogen using ethanol. The glycogen was converted into glucose using amylase enzyme (1U, Megazyme) which was measured using GOD assay (DiaSys). A control for each sample was also kept where no enzyme was added. Glycogen levels were normalized to total cellular proteins.

### Western blotting

After chronic lipotoxic treatment, myotubes were lysed and total proteins were estimated. Equal amount of protein (30 μg) was resolved by SDS-PAGE for immunoblotting. Primary antibodies used are phospho-Akt, Akt, phosphor-ERK1/2, ERK1/2, phospho-JNK, JNK, phospho-tyrosine-STAT3, phospho-serine-STAT3, STAT3, CHOP, β-actin (Cell Signaling Technology), phospho-serine-IRS1, IκB (Abcam).

For estimation of Akt phosphorylation, myotubes were washed after chronic lipotoxic treatment and then incubated in serum and glucose free medium for 1 h. Subsequently, myotubes were treated with insulin (30 nM) for 10 min and then harvested for immunoblotting.

### Measurement of cellular reactive oxygen species (ROS) level

After chronic treatments of lipotoxicity for 16 h, myotubes were loaded with DCFH-DA dye (Invitrogen) for 1 h to measure ROS. Cells were lysed and lysates were transferred to 96-black well plate to measure increase in DCF fluorescence at 485 nm excitation and 528 nm emission. Amount of ROS was normalized to total cellular DNA which was measured using bis-benzamide (Sigma) fluorescence at 360 nm excitation and 460 nm emission. Data are represented as % of control.

### Estimation of nitric oxide release

Nitric oxide release was measured in cell culture supernatant. After lipotoxic treatment, culture medium was harvested followed by centrifugation to remove debris. Culture medium was mixed with equal amount of Griess reagent (Sigma) and incubated for 15 min prior to read absorbance at 540 nm. Amount of nitric oxide release was quantified by a standard curve prepared using sodium nitrite and then normalized to total cellular proteins.

### Measurement of cytoplasmic calcium level

C2C12 myoblasts were differentiated in black 96-well plate (Corning) and were cultured under chronic lipotoxic conditions or under control conditions for 16 h. Myotubes were washed with KRBH and loaded with Fluo-4-AM, a calcium indicator fluorescent dye (Invitrogen), at 37°C for 1 h. Fluorescence readings were taken at 485 nm excitation and at 520 nm emission and expressed as arbitrary fluorescence units (AFU).

### MTT assay

After chronic lipotoxic treatment, MTT dye was added in culture medium and myotubes were further incubated for 3 h. After incubation, reduced form of MTT (formazan) was dissolved in DMSO and amounts were estimated by 560 nm absorbance after correction with 640 nm absorbance. Reduction of MTT into formazan was represented as % of control.

### Estimation of caspase-3 activity

After chronic lipotoxic treatment, myotubes were lysed in lysis buffer followed by estimation of Caspase-3 activity. Caspase-3 activity was measured by cleavage of its substrate (Ac-DEVD-pNA; Sigma) and release of pNA which was measured at 405 nm. Activity of caspase-3 (as nano-moles of pNA released per min) was normalized to total cellular protein.

### Estimation of muscle proteolysis and branched chain amino acids (BCAA) release

After chronic treatment, myotubes were washed and then incubated in KRBH for 6 h. Amount of protein secreted in KRBH and cellular protein were measured using Bradford assay (Bio-Rad). Amount of protein fragment release was normalized to total cellular proteins.

BCAA were also measured in KRBH. Samples were incubated with 100 mM glycine-KCl-KOH buffer, pH 10.5, containing 2 mM EDTA, 4 mM NAD and 6.4U of leucine dehydrogenase for 20 min. Levels of NADH formed, as a surrogate for BCAA concentration, were measured at 340 nm absorbance. Amount of BCAA release was quantified by comparing with a standard curve.

### Insulin stimulated glucose uptake

After chronic lipotoxic treatment for 16 h in the presence or absence of NAC, myotubes were starved for 30 min followed by further incubation for 30 min in KRBH containing 50 μM 2-NBDG [(2-(N-(7-Nitrobenz-2-oxa-1,3-diazol-4-yl)Amino)-2-Deoxyglucose), a fluorescent glucose analog; Invitrogen] in the presence or absence of 30nM insulin. After incubation, cells were washed twice and lysed in lysis buffer. 2-NBDG fluorescence was estimated in the lysate at 465/540 nm excitation/emission. Amount of 2-NBDG uptake was normalized with total cellular DNA which was measured using bis-benzamide (Sigma). Data are represented as % of control.

### Statistical analysis

All experiments contained four biological replicates and also repeated in separate experiments. Data are presented as mean with standard error of mean. Student’s *T*-test was performed to calculate statistical significance between control and lipotoxic conditions as represented as * P < 0.05, ** P < 0.01 and *** P < 0.001. For antioxidant treated experiments, ANOVA with Newman-Keuls post test was performed to calculate P-values among groups. Statistical significance is represented as * P < 0.05, ** P < 0.01 and *** P < 0.001.

## Abbreviations

T2DM: Type-II Diabetes mellitus
FFA: Free fatty acid
IMTG: Intramyocellular triglyceride
TG: Triglyceride
ER: Endoplasmic Reticulum
NAC: N-acetyl-L-cysteine
PDK4: Pyruvate Dehydrogenase Kinase 4
PGC1a: Peroxisome Proliferator-Activated Receptor Gamma, Coactivator 1 Alpha
HADH: 3-hydroxyacyl-CoA dehydrogenase
SDH: Succinate dehydrogenase
ROS: Reactive Oxygen Species.
